# Post-Discharge Mortality in Recently Hospitalized African Children: A Hidden Crisis

**DOI:** 10.4269/ajtmh.23-0525

**Published:** 2023-08-28

**Authors:** Chandy C. John, Davidson H. Hamer

**Affiliations:** ^1^ Ryan White Center for Pediatric Infectious Diseases and Global Health, Department of Pediatrics, Indiana University School of Medicine, Indianapolis, Indiana;; ^2^ Department of Global Health, Boston University School of Public Health, Boston, Massachusetts;; ^3^ Section of Infectious Diseases, Department of Medicine, Boston University Chobanian & Avedisian School of Medicine, Boston, Massachusetts

Hospitalized children under 5 years of age in malaria-endemic African countries have high rates of in-hospital mortality, ranging from 2–10%.^1^^,^^2^ Recent studies have shown that hospitalized children with severe anemia, much of which is attributable to malaria, also have high mortality in the post-discharge period.^3^^,^^4^ The study by Kwambai et al. in this AJTMH issue adds substantially to this body of knowledge by evaluating in-hospital and 6-month post-discharge mortality in all children under 5 years of age admitted to Siaya District Hospital, Kenya.^5^ The authors showed that, in this population, the 6-month post-discharge mortality (6.2%) was substantially higher than in-hospital mortality (2.8%), and that post-discharge mortality was particularly high in children with severe acute malnutrition (SAM, 21.6%) and severe anemia (15.5%).^5^

The study findings on post-discharge mortality were strikingly similar to those of two recently published studies. The first study, a multi-center evaluation of post-discharge mortality in Ugandan children <5 years of age with suspected sepsis, showed 6-month post-discharge mortality of 5.5%.^6^ The second study, in the Childhood Acute Illness and Nutrition (CHAIN) cohort, involving children aged 2–23 months admitted with acute illness to one of nine hospitals in Africa or Southeast Asia, showed 6–month post-discharge mortality of 5.8%.^7^ In both studies, SAM was strongly associated with increased post-discharge mortality (4.7-fold and 4.4-fold in the Ugandan and CHAIN studies, respectively), and in the Ugandan study, severe anemia was associated with a 2.7-fold increase in mortality. Other risk factors for post-discharge mortality varied across studies, but the findings from the study by Kwambai et al. add to a growing body of evidence showing that the costs and consequences of SAM and severe anemia in children <5 years of age in low-income countries extend well beyond the period of hospitalization. All three studies showed that most post-discharge deaths occur within the first 2 months of discharge, identifying this period as a time for close follow-up of recently hospitalized children. Together, the data from the 3 studies are a call to action to identify children at highest risk of death after hospitalization and to develop interventions to prevent post-discharge deaths.

In the study by Kwambai et al.,^5^ >95% of post-discharge deaths occurred in the community, so accurate diagnosis of causes of death was not possible. However, a prior study from this group found that, in children with severe anemia living in areas of moderate to high malaria transmission, post-discharge malaria chemoprevention decreased risk of death or readmission by 35% over the 6-month post-discharge period,^8^ leading to a World Health Organization recommendation that hospitalized children with severe anemia in areas of moderate to high malaria transmission receive post-discharge malaria chemoprevention.^9^ Implementation of this recommendation should contribute to a reduction in post-discharge mortality in areas of moderate to high malaria transmission, but a further understanding of the causes of death after discharge in areas across the malaria transmission spectrum, particularly in children with SAM, is needed to develop additional interventions.

The current study by Kwambai et al.^5^ serves to emphasize that SAM and severe anemia consistently predict greater risk of post-discharge death in malaria endemic African countries. In all three of the studies discussed above, standard treatment measures for SAM were followed. Thus, the findings emphasize the need for evaluation of additional areas for intervention, including those directed toward caregiver wellness and employment, which were associated with post-discharge mortality in the CHAIN study.^7^ For all pre-disposing illnesses, close follow-up, particularly in the first month post-discharge; evaluation of access to care, including potentially alerting local community health workers to the need for regular check-ins on the child; and clear instructions to care providers on warning signs or symptoms that indicate a need for urgent post-discharge medical evaluation are likely part of the solution, but multiple interventions need to be tested to see which are most effective and scalable.

Interestingly, the studies from Kwambai et al. in Kenya^5^ and Wiens et al. in Uganda^6^ both found that children with a diagnosis of malaria had a decreased risk of post-discharge mortality as compared to children without malaria. Another recent study showed that children with severe malaria had an increased risk of readmission or death as compared to community children in the same area over the same time period,^10^ so the lower risk of post-discharge mortality in children with malaria in the studies from Kwambai and Wiens likely reflects the high frequency of malaria as an admitting diagnosis, the admission of some children with non-severe malaria, and worsened outcomes when malaria is present with co-morbid conditions rather than as the sole diagnosis. Given the demonstrated efficacy of post-discharge malaria chemoprevention for children with severe anemia,^4^^,^^8^ it seems likely that malaria is playing a key role in post-discharge deaths of children with severe anemia in malaria-endemic areas. However, the three studies described above provide strong evidence that SAM is likely the greatest risk factor for post-discharge mortality in hospitalized children in low-income countries and that addressing the mortality risk will require nutrition- and family-focused interventions beyond post-discharge malaria chemoprevention.

Ultimately, if we are to achieve WHO Sustainable Development Goal Target 3.2 to “end preventable deaths of newborns and children under 5 years of age”,^11^ additional research on causes of death post-discharge, and consequent development of additional post-discharge interventions, is urgently needed.
